# Prevalence of death anxiety and its related factors in the population of eastern Iran: a cross-sectional study in the era of COVID-19

**DOI:** 10.3389/fpubh.2024.1427995

**Published:** 2024-11-27

**Authors:** Hossein Bakhtiari-Dovvombaygi, Mohammadreza Askari, Mohammad Rahimkhani, Mahboobeh Abdollahi, Mohammadreza Baladastian, Amir Alipour, Mohammad Namazinia

**Affiliations:** ^1^Student Research Committee, Torbat Heydariyeh University of Medical Sciences, Torbat Heydariyeh, Iran; ^2^Student Research Committee, School of Nursing and Midwifery, Shahid Beheshti University of Medical Sciences, Tehran, Iran; ^3^Department of Nursing, School of Nursing and Midwifery, Torbat Heydariyeh University of Medical Sciences, Torbat Heydariyeh, Iran; ^4^Department of Biostatistics, School of Health, Torbat Heydariyeh University of Medical Sciences, Torbat Heydariyeh, Iran; ^5^Department of Nursing, School of Nursing and Midwifery, Iran University of Medical Sciences, Tehran, Iran; ^6^Student Research Committee, Sabzevar University of Medical Sciences, Sabzevar, Iran

**Keywords:** death anxiety, COVID-19, epidemiology, mental health, pandemics

## Abstract

**Background:**

The global COVID-19 pandemic has triggered widespread anxiety, including a significant rise in death anxiety. If unaddressed, death anxiety can lead to numerous mental and physical health issues. This study aimed to assess the prevalence of death anxiety and its associated factors in the population of eastern Iran.

**Methods:**

This cross-sectional study included 515 participants from Torbat Heydariyeh in 2019. Data were collected using a structured two-part questionnaire: demographic data and the Templer Death Anxiety Scale. Sampling was conducted through a stratified approach to represent the city's population distribution accurately.

**Results:**

The mean age of participants was 32.88 ± 10.75 years (range: 18–80). The mean death anxiety score was 6.72 ± 3.11. Multiple linear regression analysis revealed significant associations between death anxiety and gender (B = 1.12, β = 0.23, *P* = 0.001), education level (B = 1.75 for high school or lower, β = 0.19, *P* < 0.05), smoking (B = 0.76, β = 0.15, *P* = 0.049), and economic status (B = −0.82, β = −0.12, *P* = 0.006). No significant relationship was found between death anxiety and age, marital status, occupation, medical history, medication use, health status, or income source (*P* > 0.05).

**Conclusions:**

This study demonstrates that participants exhibited moderate levels of death anxiety. Given its potential to impact quality of life, strategies for managing death anxiety should be considered in similar public health crises.

## Introduction

In December 2019, the COVID-19 outbreak began in Wuhan, China, causing widespread concern worldwide. In just two and a half months, the World Health Organization declared it a global pandemic due to the rapid spread and severe impact of the virus (1). The uncertainty and unpredictability surrounding this disease, combined with strict health measures like quarantines and reduced services, have led to significant stress and anxiety globally (2–4, 41, 42). One critical psychological consequence of such stressful events, particularly life-threatening illnesses like COVID-19, is death anxiety (3).

Death anxiety is defined by the British National Health Service as a feeling of panic or extreme worry when contemplating death, the process of dying, or what happens afterward (5). This anxiety can disrupt an individual's normal functioning, resulting in severe psychological distress (6–8). The negative effects of death anxiety may include increased pessimism, hopelessness, diminished social support, and physical health problems (9).

Furthermore, cultural values strongly influence how different populations experience death anxiety. Understanding these cultural differences is crucial for addressing the psychological impacts of pandemics and informing health interventions (10, 11). In Iran, the COVID-19 pandemic has had a significant impact on mental health, with high levels of stress and anxiety reported among the general population (12–14). Those who were more frequently exposed to media and social media during the pandemic experienced even greater anxiety (4, 15, 16, 43, 44). Death anxiety, as a potent emotional response, has been noted to increase as people face the imminent risk of losing their lives during pandemics (17). For example, a study in Shahroud, Iran, reported moderate levels of death anxiety during the COVID-19 pandemic (18), and similar findings were observed in Lebanon (10).

### Research problem and justification

Despite the considerable attention given to mental health issues during the COVID-19 pandemic, there is limited research specifically addressing death anxiety in eastern Iran, a region with distinct socio-cultural and economic features (19). Eastern Iran is more traditional and less urbanized than the western and central parts of the country, which may result in unique psychological responses to the pandemic (20). Furthermore, the region's public health infrastructure, which may be less developed and less equipped to handle mental health crises, could exacerbate these challenges. Investigating death anxiety in this region is essential for tailoring mental health interventions and public health policies to the local population's needs (21).

Given the lack of research in eastern Iran, this study was conducted to determine the prevalence of death anxiety and its related factors within this population. While the study took place in 2019, the findings remain relevant as COVID-19 continues to impact mental health worldwide. This study's results can provide insight into how death anxiety manifests during pandemics, offering valuable lessons for future health crises and their psychological consequences.

## Methods

### Study design and study setting

The current study is a cross-sectional study that was conducted in 2019 on 515 residents of Torbat Heydariyeh city. The research community was all the people of Torbat Heydariyeh city.

### Participants

The inclusion criteria for the study were:

- Providing informed consent,
- Being at least 18 years old,
- Residing in Torbat Heydariyeh city at the time of the study.

### Sample size and randomization

To determine the sample size, we utilized a sample size calculation formula for estimating proportions, specifically the formula:


$$\text{n=}({\text{Z}}^{2}\;*\text{ p }*\;(\text{1- p})){\text{/d}}^{2}$$

where:

*n* = required sample size
Z = Z-value (1.96 for a 95% confidence level)
*P* = estimated proportion of the population (0.5 was used for maximum variability)
d = margin of error (set at 0.05).

Based on this formula, the estimated sample size was calculated to be 515 participants. Randomly stratified and weighted sampling was then conducted based on the population distribution in each area of the city. The city was divided into five parts (North, South, East, West, and Center); the number of samples required for each region was calculated in proportion to the overall sample size of the study, ensuring adequate representation from each area.

Efforts to Reduce Selection Bias:To minimize selection bias, the sampling method was meticulously designed to ensure that the sample accurately reflected the population of Torbat Heydariyeh. The random stratified sampling approach took into account demographic and geographical differences across the five parts of the city, thus providing a balanced representation of all subgroups within the population.

### Outcomes measures

The data collection instrument consisted of two parts. The first part was demographic questions, including age, gender, education, marital status, occupation, medical history, health status, economic status, and source of income. In the second part, there were questions related to death anxiety. Templer's Death Anxiety Scale (DAS) was used to evaluate death anxiety. This questionnaire included 15 Yes/No questions, where a score of one was given for “yes” and zero for “no” (a score of zero indicates the absence of death anxiety and a score of one indicates the presence of death anxiety). The total scores ranged from 0 (no death anxiety) to 15 (very high death anxiety) (15 Death anxiety scores were classified into three levels: mild anxiety (0–6), moderate (7–9), and severe (10–15). Templer's death anxiety scale is a standard questionnaire used worldwide and was translated and psychometrically evaluated in Iran by Rajabi et al. in (40). The reliability coefficient of classification was 0.6, and the internal consistency coefficient was 0.73 (16). Thomas et al. recorded its reliability as 0.76 and its internal consistency as 0.83 using the test-retest method (17). In the present study, the reliability of the questionnaire was calculated with a Cronbach's alpha coefficient of 0.75.

### Data collection

Due to the spread of the COVID-19 disease and in compliance with health guidelines, face-to-face interviews were avoided. With the cooperation of the Torbat Heydariyeh City Telecommunications Department, the landline phone numbers for each region were randomly obtained. The researcher made a phone call to the research units, introducing themselves, explaining the objectives of the research, ensuring the confidentiality of the information, and obtaining informed consent. The researcher then read and completed the questionnaire questions without any additional explanation.

Handling Missing Data: To address missing data, we employed a complete case analysis approach, ensuring that only participants with fully completed questionnaires were included in the analysis. Furthermore, a sensitivity analysis was performed to ensure that excluding missing data did not introduce significant bias into the results. Any significant missing data patterns were assessed and reported accordingly.

### Statistical analysis

Data analysis was performed using the 16th version of SPSS software. The frequency (percentage) was used to describe qualitative data, and the mean ± standard deviation was used for quantitative data. To ensure the validity of the analysis, several statistical assumptions were tested before applying the regression models. The Kolmogorov-Smirnov test was used to check for the normality of the data distribution. Additionally, multicollinearity between independent variables was examined using the Variance Inflation Factor (VIF), ensuring that no significant collinearity existed. The decision to use multiple linear regression was made based on its appropriateness for investigating the relationship between the dependent variable (death anxiety) and multiple independent variables. This method allowed for the adjustment of confounding variables to isolate the effect of each factor on death anxiety.

Confounding Variables: The analysis adjusted for several potential confounders, including age, gender, education, marital status, occupation, health status, and exposure to COVID-19 news. These variables were included in the regression models to control for their potential influence on death anxiety.

## Results

The average age of participants was 32.88 ± 10.75 years, with a range from 18 to 80 years. Most participants, 357 (69%), were married, and 339 people (66%) were satisfied with their economic situation. The average number of children was 1.20 ± 1.25, and the majority of participants, 321 (62%), reported their health status as good, while the remaining participants reported it as average. Further demographic information is presented in Table 1. Most participants, 65%, had no history of illness, with the frequency of illnesses detailed in Table 2. Additionally, 448 participants (87%) reported that they do not smoke (Table 3).

**Table 1:** Demographic information of people participating in the study.

**Variable**	**N (%)**
Age (Mean ± SD)	32.88 ± 10.75
**Gender**	
Female	250 (49%)
male	265 (51%)
**Education**	
Lower than high school	61 (12%)
High school	143 (25%)
University education	311 (60%)
**Occupation**	
Employed in governmental organizations	188 (36%)
Housewife	114 (22%)
Student	91 (18%)
Non-governmental jobs	122 (24%)
**Medical history**	
Yes	182 (35%)
No	333 (65%)
**Taking any drugs**	
Yes	132 (26%)
No	383 (74%)
**Smoking**	
Yes	67 (13%)
No	444 (87%)
**Source of income**	
Family	244 (47%)
Myself	271 (53%)

**Table 2:** Frequency of diseases of people participating in the study.

**Type of disease**	***N* (%)**
Psychological	6 (1%)
Cardiovascular or blood pressure	32 (7%)
Endocrinologic	21 (4%)
Neurologic	17 (3%)
Hepatic or digestive	28 (6%)
Nephrological or urologic	17 (3%)
Skin	6 (1%)
Respiratory	22 (4%)
Musculoskeletal	12 (2%)
Other	21 (4%)

**Table 3:** Prevalence of smoking among the participants.

**Type of tobacco consumed**	***N* (%)**
Hookah	34 (7%)
Cigarette	26 (5%)
Traditional tobacco products	7 (1%)

The results indicated that the average death anxiety score was 6.73 ± 3.11, with scores ranging from 0 to 14. Most participants, 244 (47%), had mild death anxiety, while 168 (33%) had moderate death anxiety, and 103 people (20%) had severe death anxiety. The Kolmogorov-Smirnov test confirmed that the distribution of death anxiety scores followed a normal distribution (*P* = 0.11). Therefore, multiple linear regression was employed to analyze factors related to death anxiety (Table 4).

**Table 4:** Factors related to death anxiety.

**Variable**	**Regression coefficient**	***P* value**	**Confidence interval 95%**
**Gender**			
Female	1.12	0.001	0.47, 1.76
Male	-	-	-
Married	−0.17	0.66	−0.93, 0.59
Single	-	-	-
**Education level**	
Lower than high school	1.75	<0.001	0.82, 2.68
High school	0.34	0.299	−0.30, 0.97
University education	-	-	-
**Smoking**	
Yes	0.76	0.049	0.08, 1.61
No	-	-	-
**Satisfied with economic status**	
Yes	−0.82	0.006	−0.1.41, −0.24
No	-	-	-

The regression analysis revealed several key factors associated with death anxiety:

Gender: Women had an average death anxiety score 1.12 points higher than men (*P* = 0.001).

Education Level: People with a high school diploma or lower had significantly higher death anxiety scores compared to those with a university education. The difference was 1.75 points for high school graduates and 0.34 points for those with some higher education (*P* < 0.05).

Smoking Status: Smokers had an average death anxiety score 0.76 points higher than non-smokers (*P* = 0.049).

Economic Satisfaction: Participants who were satisfied with their economic status had an average death anxiety score 0.82 points lower than those who were not satisfied (*P* = 0.006).

No statistically significant relationship was observed between death anxiety and variables such as age, marital status, occupation, history of illness, medication use, health status, source of income, or number of children (*P* > 0.05).

## Discussion

This study aimed to assess the prevalence of death anxiety and its associated factors in the population of eastern Iran, finding that death anxiety related to COVID-19 was moderate. Our findings revealed that age was not significantly associated with death anxiety, which is consistent with the study by Aghajani et al. (22), but contradicts the results of Henrie et al. (23) and Soleimani et al. (24). Before the pandemic, younger individuals may not have considered death as much as older adults. However, the universal risk posed by COVID-19 may have influenced people of all ages similarly, explaining the lack of significant correlation between age and death anxiety in this study.

A significant relationship between gender and death anxiety was observed, with women reporting higher levels of anxiety than men. This finding aligns with the results of Thorson et al. (25), Pierce et al., and Mansurnezhad et al. (26). This difference can be attributed to both emotional and cultural factors, as women may be more expressive about their anxieties and concerns regarding death compared to men (27, 28). However, this outcome contrasts with the findings of Mousavi et al. (29), indicating that further investigation into gender differences in death anxiety is warranted.

Regarding economic status, individuals with lower economic standing experienced higher death anxiety than those with better economic conditions, supporting the conclusions of Misler et al. (30). Those with greater access to financial resources and services may be better equipped to cope with anxiety, reducing their overall stress levels. These findings align with Soleimani et al. (24) and Chang et al. (31), though studies such as Postolica et al. (32) and Assari et al. (33), reported the opposite, with individuals of higher economic status exhibiting more death anxiety. This discrepancy may stem from differences in how economic resources are perceived in various cultural contexts.

Education also played a crucial role, with university-educated individuals reporting lower death anxiety than those with lower levels of education. This finding is consistent with the results of Azaiza et al. (34), who noted that higher education levels contribute to increased awareness and understanding of preventive measures against COVID-19, thereby reducing anxiety.

Although marital status did not significantly influence death anxiety, married individuals exhibited slightly higher anxiety scores than their single counterparts. This result aligns with studies by Rouhi et al. (35) and Massoudzadeh et al. (36), though Aghajani et al. (22) found that single people experienced more death anxiety.

Occupation was another key factor influencing death anxiety. Medical professionals exhibited the lowest levels of anxiety, likely due to their familiarity with health-related risks, while retirees reported the highest levels of anxiety. This could be attributed to age-related factors, chronic health conditions, and financial insecurity among retirees (37, 38).

Finally, a significant relationship between source of income and death anxiety was observed. Participants reliant on charitable organizations or support institutions reported higher levels of anxiety, likely due to financial dependence and concerns over healthcare expenses during the pandemic. This finding contrasts with the study by Soleimani et al. (39), indicating a need for further research on how income sources impact psychological wellbeing.

Despite these important findings, the study has several limitations. The cross-sectional design limits the ability to establish causal relationships, as the data only reflect associations at one point in time. Longitudinal studies are recommended to explore the temporal relationships between death anxiety and its associated factors.

Additionally, the generalizability of the findings is limited by the study's regional focus. While the study provides valuable insights into the population of eastern Iran, its applicability to other regions or populations must be approached with caution. Cultural, social, and public health differences may significantly influence death anxiety across different regions. Future studies in diverse geographic and pandemic contexts are needed to improve external validity and ensure broader applicability.

One significant limitation was the digital divide in the studied population, as unequal access to online services may have affected the representativeness of the sample. Some individuals, particularly those in rural or underserved areas, may have been excluded due to limited access to internet or telephone services, potentially introducing bias into the results. To adhere to health guidelines during the pandemic, data collection was conducted virtually, which may have further limited participation for certain groups.

In future research, employing alternative data collection methods, such as in-person interviews or a mixed-methods approach, could improve.

## Conclusion

The results of this study indicate that the level of death anxiety among the population of Torbat Heydariyeh city during the COVID-19 pandemic was moderate. Given that death anxiety was present across various demographic groups, including individuals of different ages, marital statuses, and health backgrounds, it is crucial to implement educational programs aimed at teaching effective coping strategies. Additionally, it is essential to recognize the role of cultural and social factors in shaping death anxiety, as these may vary across different geographic regions. Therefore, conducting further research in diverse populations is recommended to develop a more comprehensive understanding of death anxiety and to support the development of targeted interventions.

## Data Availability

The raw data supporting the conclusions of this article will be made available by the authors, without undue reservation.
